# GIWAXS using microbeam applied in halide perovskite films for high spectral resolution and mapping capability

**DOI:** 10.1107/S1600577526004303

**Published:** 2026-05-21

**Authors:** Meirong Fu, Bingchen He, Liujiang Zhang, Zhenhuang Su, Yanfeng Miao, Qihang Sun, Jihao Zhang, Sisheng Wang, Zhijun Wang, Xingya Wang, Yuzhu Wang, Bo Sun, Wen Wen, Yixin Zhao, Chenyue Wang, Xingyu Gao

**Affiliations:** a Shanghai Institute of Applied Physics Chinese Academy of Sciences Shanghai201800 People’s Republic of China; b Shanghai Synchrotron Radiation Facility, Shanghai Advanced Research Institute Chinese Academy of Sciences Shanghai201204 People’s Republic of China; c University of Chinese Academy of Sciences Beijing100049 People’s Republic of China; d The Institute for Advanced Studies Wuhan University Wuhan430072 People’s Republic of China; e School of Environmental Science and Engineering, Frontiers Science Center for Transformative Molecules Shanghai Jiao Tong University Shanghai200240 People’s Republic of China; University of Malaga, Spain

**Keywords:** perovskite, µ-GIWAXS, radiation damage, mapping, footprint effect

## Abstract

Synchrotron-based grazing-incidence wide-angle X-ray scattering (GIWAXS) has become an essential tool for investigating halide perovskite film microstructures. Nevertheless, GIWAXS employing 100 µm X-ray beams inevitably suffers from footprint effects manifesting as diffraction peak broadening and spectral distortions. Herein, we demonstrate that microbeam GIWAXS (µ-GIWAXS) effectively mitigates these footprint effects, achieving enhanced spectral resolution and accuracy. By systematically tuning the grazing incidence angle, we quantitatively illustrated how the footprint length contributes to diffraction peak broadening in µ-GIWAXS. In addition, we optimize µ-GIWAXS data acquisition through beam-direction-perpendicular fly scanning, which improves data quality while preserving resolution and minimizing radiation damage at individual spots. Through extended exposure experiments, the effect of µ-GIWAXS microbeam irradiation on the structural stability of perovskite films was systematically evaluated. Furthermore, µ-GIWAXS enables spatial mapping of perovskite film microstructural characteristics including crystallinity, preferential crystal orientation, PbI_2_ concentration and lattice strain – critical information for addressing inhomogeneity challenges in large-area perovskite devices. Given the growing demand in scalable perovskite optoelectronic applications, µ-GIWAXS warrants increased attention due to its dual advantages of superior spectral resolution and spatially resolved analysis capabilities.

## Abstract 2

This article demonstrates that microbeam GIWAXS (µ-GIWAXS) effectively overcomes the distorting footprint effects of conventional GIWAXS analysis of perovskite films. This advancement not only delivers higher reciprocal-space resolution data but also unlocks the unique capability to spatially map microstructural inhomogeneity, which is vital for the scalable manufacturing of perovskite optoelectronics.

## 1. Introduction

Metal halide perovskites have emerged as a research focus in optoelectronic fields (Zhang *et al.*, 2023 ▸; Song *et al.*, 2025 ▸; Sakhatskyi *et al.*, 2023 ▸) due to their strong light absorption, high carrier mobility, low cost, and low-temperature solution processability (Wei *et al.*, 2024 ▸; Duan *et al.*, 2024 ▸). Single-junction perovskite solar cells (PSCs) have already reached a certified power conversion efficiency (PCE) of 27.3%, rivaling the performance of commercial crystalline silicon photovoltaics (see https://www.nrel.gov/pv/interactive-cell-efficiency.html). In addition, perovskite–silicon tandem devices also have reached a PCE of 35.0%, highlighting their great potential for commercialization (Aydin *et al.*, 2024 ▸). The microstructural characteristics of perovskite films are universally recognized as critical determinants of device performance, a subject of extensive investigation in recent years (Zhou *et al.*, 2022 ▸; Guo *et al.*, 2024 ▸). Among various characterization techniques, synchrotron-based grazing-incidence wide-angle X-ray scattering (GIWAXS)/grazing-incidence X-ray diffraction (GIXRD) has emerged as one of the most widely applied techniques to investigate the microstructures of perovskite films (Liang *et al.*, 2023 ▸). Recent years have witnessed a rapid increase in GIWAXS-based studies of PSCs (Park *et al.*, 2023 ▸; Li, Su *et al.*, 2023 ▸; Huang *et al.*, 2023 ▸).

In GIWAXS measurements, the diffraction signals are collected from the entire X-ray illuminated region on the sample surface, which is significantly expanded by the so-called footprint effect at grazing incident angles. This geometric effect advantageously increases the X-ray–sample interaction volume at the surface, contributing to GIWAXS’s widespread application in thin film characterization. While surface sensitivity can be enhanced by reducing the grazing angle, this comes at the cost of further elongating the X-ray footprint. However, at synchrotron facilities dedicated to X-ray diffraction/scattering techniques, GIWAXS is typically conducted with beam sizes ranging from hundreds of micrometres. Consequently, the footprint can exceed common sample dimensions at extreme grazing angles, leading to peak broadening since X-ray diffraction peaks originating from different positions along the elongated footprint project as overlapping signals on the area detector (Smilgies, 2009 ▸). Furthermore, both non-uniform beam intensity profiles and sample microstructural inhomogeneities may introduce distortions in the diffraction patterns.

An effective strategy to mitigate this issue involves replacing conventional millimetre-scale beams with microbeams or even nanobeams in GIWAXS experiments. The elongated footprint thus becomes much shorter, thereby narrowing the diffraction peak distribution projected onto detectors. In fact, microbeam X-ray diffraction (µ-XRD) or even nanobeam XRD (n-XRD) has been proven successful in analyzing small samples or a small area/volume of samples (Goldsmith & Walker, 1983 ▸; Nozaki *et al.*, 2020 ▸). By combining high-brilliance synchrotron sources with advanced X-ray focusing optics, sub-micrometre polychromatic/monochromatic beams can be achieved for spatial mapping of grain orientation, strain/stress, and micro-topography (Li *et al.*, 2022 ▸). For example, scanning n-XRD in transmission geometry was used to simultaneously probe the morphology and the structural properties of spin-coated MAPbI_3_ perovskite films (Lilliu *et al.*, 2016 ▸). Nevertheless, µ-XRD/n-XRD does not take intrinsic advantages of grazing incidence ideal for detecting thin films. For such purposes, a few GIXRD experiments, using a beam with a diameter of only a few micrometres (µ-GIXRD), have been reported to investigate structurally inhomogeneous samples (Sáez-Martínez *et al.*, 2021 ▸). Recently, the application of µ-GIWAXS has been successfully introduced to halide perovskite films, demonstrating its powerful capability for spatially resolved microstructural mapping (Chan *et al.*, 2025 ▸; Zhang *et al.*, 2025 ▸). However, a systematic investigation of the fundamental aspects of µ-GIWAXS—including the quantitative relationship between beam footprint and spectral broadening, optimized data acquisition strategies, radiation damage assessment under high-flux-density microbeam and comprehensive multi-parameter spatial mapping—remains lacking.

In this study, µ-GIWAXS/µ-GIXRD measurements of halide perovskite films were explored by using both an X-ray beam with vertical size of about 3 µm at the BL03HB beamline and that of about 1 µm at the BL17UM beamline of the Shanghai Synchrotron Radiation Facility (SSRF). Parallel GIWAXS measurements with a beam size of about 300 µm were simultaneously conducted at BL14B1 for direct comparison. Unless otherwise specified, beam sizes are defined as the full width at half-maximum (FWHM). The experimental results demonstrate significantly reduced footprint effects in µ-GIWAXS spectra, yielding enhanced spectral resolution. Additionally, scanning µ-GIWAXS was implemented to spatially resolve microstructural heterogeneities across perovskite films. Given that these local microstructural variations are known to influence the long-term stability and efficiencies of large-area perovskite devices, µ-GIWAXS represents an effective and valuable tool for accelerating their commercial development (He *et al.*, 2023 ▸; Jiang *et al.*, 2025 ▸; Yin *et al.*, 2025 ▸).

## 2. Material and methods

### 2.1. Materials

All the raw materials were purchased from commercial suppliers without further purification. Patterned indium tin oxide (ITO) glass was purchased from Advanced Election Technology Co. Ltd. *N*,*N*-di­methyl­formamide (DMF), di­methyl sulfoxide (DMSO), chloro­benzene (CB) and iso­propanol (IPA) were purchased from Sigma Aldrich. Lead iodide (PbI_2_) was purchased from TCI. Formamidinium iodide (FAI), methyl­ammonium chloride (MACl), methyl­ammonium iodide (MAI) and caesium iodide (CsI) were all purchased from Xi’an Polymer Light Technology. Except for ITO glass, all the materials were stored in a nitro­gen-filled glove box to avoid water.

### 2.2. Perovskite precursor and films preparation

ITO glass was ultrasonically cleaned by sequentially washing with detergent, deionized water, ethyl alcohol and iso­propanol (IPA). Before use, the cleaned ITO substrates (with lateral dimensions of 15 mm × 20 mm) were dried using a nitro­gen gun and then treated with ultraviolet ozone for 20 min to improve the surface wettability of the surfaces. For the pure MAPbI_3_ film preparation, PbI_2_ (461 mg) and MAI (159 mg) in DMF:DMSO = 700 µL:78 µL was stirred for 2 h and then filtered with a 0.22 µm polytetra­fluoro­ethyl­ene (PTFE) filter. 40 µL of MAPbI_3_ precursor was spin-coated at 4000 rpm for 30 s (4 s acceleration to 4000 rpm). 100 µL CB was dropped on the film at 22 s before the end of the spin-coating. The film was annealed by 100 °C hot plate in nitro­gen glovebox for 10 min. For the preparation of the mixed hybrid Cs_0.05_FA_0.85_MA_0.1_PbI_3_ (hereafter denoted as CsFAMAPbI_3_ for brevity) film, a precursor solution was first prepared by dissolving PbI_2_ (691.5 mg) and CsI (19.5 mg) in a solvent mixture of DMF and DMSO in a 900 µL:100 µL ratio. The solution was stirred for 1 h and subsequently filtered using a 0.22 µm PTFE filter. A 40 µL aliquot of the PbI_2_ solution was spin-coated onto ITO glass at 1500 rpm for 30 s, followed by annealing at 70 °C for 1 min in a nitro­gen atmosphere. After cooling to room temperature, 80 µL of a pre-filtered organic salt solution—comprising FAI:MAI:MACl in a weight ratio of 90 mg:6.39 mg:9 mg dissolved in 1 ml of iso­propanol—was spin-coated onto the PbI_2_ layer at 2000 rpm for 30 s. The resulting film was then transferred from the nitro­gen glovebox to a hot plate in ambient air (30–40% relative humidity) and annealed at 150 °C for 15 min.

### 2.3. GIWAXS characterization

GIWAXS measurements were performed at the BL03HB, BL14B1 and BL17UM beamlines of the SSRF, China (Xing-Yu *et al.*, 2015 ▸; Wang, Wang *et al.*, 2024 ▸; Kong *et al.*, 2025 ▸). The key experimental parameters are summarized in Table 1 ▸ and are described below.

BL14B1 is a bending magnet beamline (1.27 T) equipped with a collimating mirror, a focused Si(111) double-crystal monochromator (DCM), and a vertically focusing mirror. No micro-focusing optics are employed; the beam is shaped by a slit system to approximately 300 µm (V) × 500 µm (H) (FWHM) at the sample position, with an energy resolution of Δ*E*/*E* ≃ 1.91 × 10^−4^ and a beam divergence of ∼0.25 mrad (V). The intrinsic beamline divergence and the divergence at the sample position are essentially identical, as no focusing optics are used. A Huber 5021 six-circle diffractometer provides multi-axis sample positioning. The 2D GIWAXS patterns were recorded using a MarCCD225 detector (pixel size ≃ 73 µm × 73 µm) at a sample-to-detector distance of ∼250 mm.

BL03HB is a Laue microdiffraction beamline based on a superbend dipole magnet (2.29 T), featuring a two-stage focusing design. The optical system comprises a toroidal mirror for horizontal pre-focusing, a four-bounce Si(111) monochromator that can switch between monochromatic and white-beam modes, and a vertically focusing mirror that creates a secondary source. Two sets of Pt-coated Kirkpatrick–Baez (KB) mirrors are installed downstream: the first set (at 46 m from the source) focuses the beam to ∼4 µm × 4 µm in the protein crystallography sector, and the second set (at 51 m) further demagnifies the beam. The µ-GIWAXS measurements in this work were conducted in the materials science sector using the monochromatic mode at 10 keV (Δ*E*/*E* ≃ 0.96 × 10^−4^), with a beam size of approximately 3 µm (V) × 5 µm (H) at the sample position. Due to the large numerical aperture required for KB micro-focusing, the effective beam divergence at the sample position (∼2.8 mrad) is substantially larger than the intrinsic beamline divergence upstream of the KB system. The 2D µ-GIWAXS patterns were recorded using a Pilatus 2M area detector (pixel size 172 µm × 172 µm) at a sample-to-detector distance of ∼250 mm. A six-degrees-of-freedom sample stage (three translational and three rotational motions) enables precise positioning and alignment.

BL17UM is a high-performance undulator beamline (CPMU20, 20 mm period, 160 periods) designed for micro-crystallography. The beamline features interchangeable optics: a cryo-cooled Si(111) DCM (Δ*E*/*E* ≃ 2 × 10^−4^) for high-resolution applications and a double multilayer monochromator (DMM) for high-flux experiments. The beam is focused by a pair of KB mirrors to approximately 1 µm × 0.7 µm (FWHM) at the sample position, with an effective divergence of ∼2.5 mrad (H) × ∼1.0 mrad (V). In this work, the DCM mode at 10 keV was used for µ-GIWAXS measurements. The 2D patterns were recorded using an Eiger X 16M detector (pixel size 75 µm × 75 µm) at a sample-to-detector distance of ∼300 mm. An MD3-UP diffractometer is used to control precise positioning and alignment.

At all of the beamlines, the diffraction spectra were calibrated using NIST SRM660b (LaB_6_) sample. The GIWAXS/µ-GIWAXS data were processed by using MATLAB and *Fit2d* software.

## 3. Results and discussion

### 3.1. Comparison of µ-GIWAXS and GIWAXS

**Figure 1:**
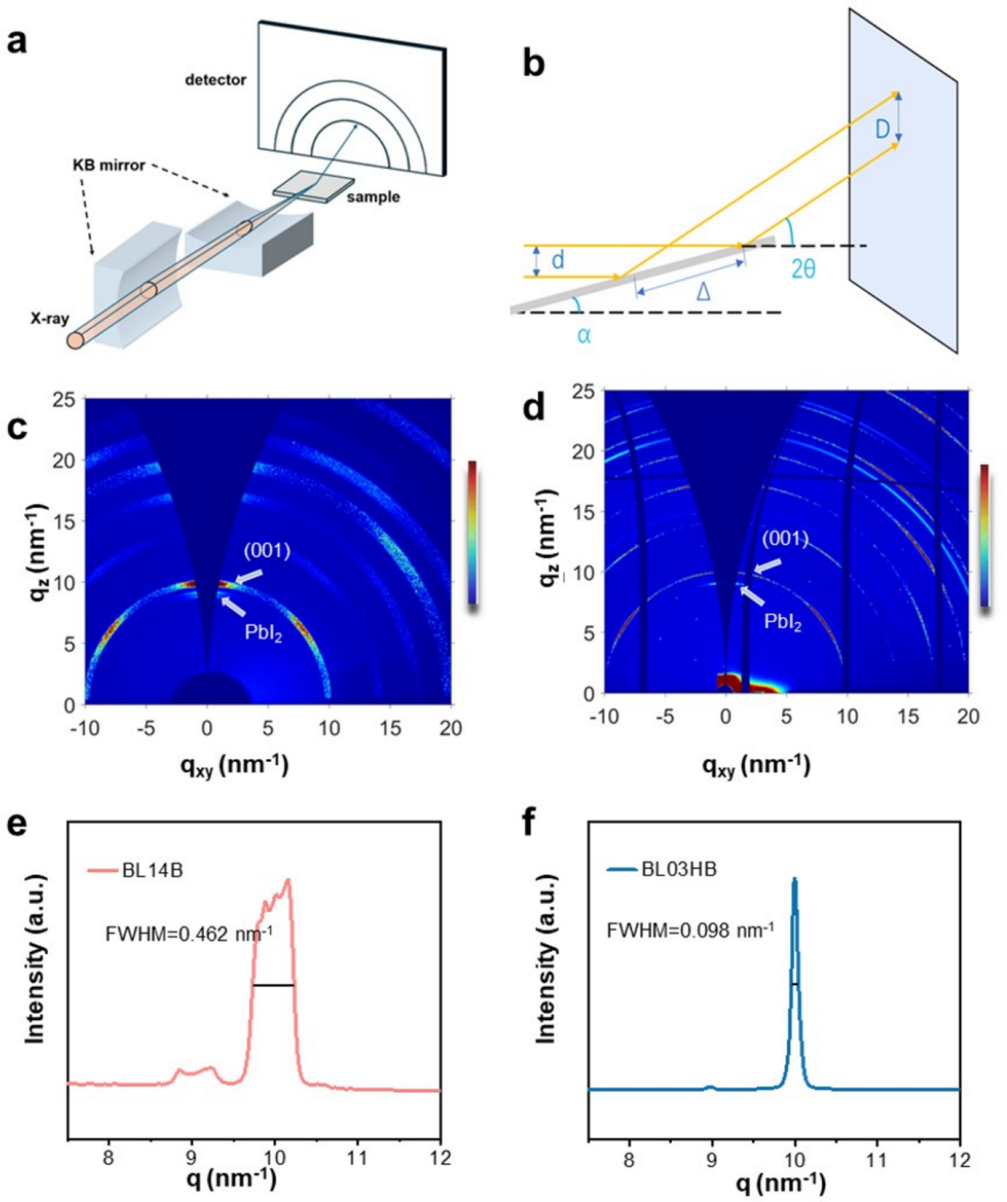
(*a*) Schematic of X-ray microfocusing and the µ-GIWAXS experimental setup. (*b*) Schematic illustrating the broadening of a diffraction peak in GIWAXS due to the footprint effects. 2D GIWAXS patterns of a CsFAMAPbI_3_ perovskite film measured with an incidence angle of 1° at the beamline BL14B1 in (*c*) and BL03HB in (*d*), respectively. The corresponding 1D GIWAXS diffraction spectra derived from (*c*) and (*d*) are shown in (*e*) and (*f*), respectively.

Fig. 1 ▸(*a*) presents the µ-GIWAXS geometry featuring a set of KB mirrors for X-ray micro-focusing. To quantify footprint effects, Fig. 1 ▸(*b*) models a grazing-incidence (α) X-ray beam (of diameter *d*) generating an out-of-plane diffraction peak at 2θ onto the area detector with a vertical projection of *D*. From geometric analysis, the footprint in Fig. 1 ▸(*b*) is deduced to be Δ = *d*/sinα with the projection 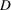 = 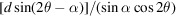. The beam diameters at the BL14B1 and the BL03HB beamlines are approximately 300 µm and 3 µm, respectively. At an incidence angle of 1° for a diffraction angle of 11.33° [corresponding to the perovskite (001) reflection at a photon energy of 10 keV], the Δ values at the BL14B1 and the BL03HB beamlines are 17.19 mm and 171.9 µm with a *D* of 3143.66 µm and 31.44 µm, respectively. Considering the significant beam divergence angle resulting from KB mirror focusing at the BL03HB beamline, we have incorporated the contribution of beam divergence into our analysis (Fig. S1 of the supporting information). Under identical conditions, we further incorporated the specific beam divergence angles of the two beamlines: β_1_ = 0.25 mrad for BL14B1 and β_2_ = 2.8 mrad for BL03HB. A typical sample-to-detector distance of 250 mm was used. Substituting these values into *D* = 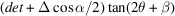 − 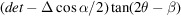, the revised *D* at the BL14B1 and the BL03HB beamlines are calculated as 3573.68 µm and 1486.76 µm, respectively. These results clearly demonstrate that the use of a large beam size leads to considerable diffraction peak broadening, due to the increased beam projection. It should be noted that this estimation of instrumental broadening arising from footprint effects is simplified and does not account for the intrinsic diffraction peak width. The observed peak width is also determined by the natural diffraction profile, which is governed by the microstructural properties of the films, including Miller indices, grain size, strain, defects and preferred orientation (Holder & Schaak, 2019 ▸).

Beyond the footprint contribution, the instrumental broadening of the peak is a convolution of several additional factors. First, the beam divergence at the sample position—arising from the combined contributions of the intrinsic source emittance and the focusing optics—broadens diffraction peaks along the radial direction. This specific contribution has been explicitly incorporated into our projection analysis above. Second, the detector point-spread function (PSF) and the finite pixel sizes of the detectors used in this work impose a fundamental lower limit on the achievable angular resolution at a given sample-to-detector distance (Smilgies, 2009 ▸). Third, the geometric projection of scattering from the curved Ewald sphere onto a flat planar detector introduces a non-linear distortion of diffraction rings that exacerbates at higher scattering angles, contributing to additional apparent broadening (Jiang, 2015 ▸). To mitigate this, the detectors were positioned approximately normal to the direct beam, and rigorous geometric corrections—accounting for detector tilt and sample-to-detector distance uncertainty—were applied during data reduction using LaB_6_ calibration. Any error in this calibrated sample-to-detector distance would propagate directly into the *q*-vector scale, introducing systematic shifts and artificial broadening in the integrated profiles. Furthermore, the finite energy bandwidth (Δ*E*/*E* ≃ 10^−4^) of the Si(111) monochromator produces a wavelength spread that broadens peaks proportionally to tanθ (Birkholz, 2006 ▸). Finally, the integration geometry during data reduction (*e.g.* azimuthal integration) introduces geometric smearing when curved diffraction rings are binned over a finite azimuthal angle onto a Cartesian grid (Jiang, 2015 ▸). Among these contributions, the beam footprint and beam divergence are the dominant instrumental factors in the present GIWAXS/µ-GIWAXS measurements, while the remaining contributions are comparatively minor but are accounted for in the data reduction procedure.

To systematically investigate the influence of footprint effects on GIWAXS measurements in perovskite films, we conducted comparative experiments on CsFAMAPbI_3_ samples using two different beamline setups: BL14B1 and BL03HB. The GIWAXS diffraction patterns collected at the same photon energy of 10 keV with an incidence angle of 1° are presented in Figs. 1 ▸(*c*) and 1 ▸(*d*), respectively. This incidence angle was chosen to ensure that the standard beam footprint remains within the sample boundaries; at smaller grazing angles the footprint would exceed the sample dimensions, convolving footprint broadening with sample-edge truncation effects (Pflüger *et al.*, 2017 ▸). The intense scattering feature near the beamstop arises from specular reflection, Yoneda diffuse scattering at the critical angle (α_c_), and parasitic scattering, and is confined to the low-*q* region (*q* < ∼5 nm−^1^), well separated from the perovskite and PbI_2_ reflections of interest (Yoneda, 1963 ▸; Hexemer & Müller-Buschbaum, 2015 ▸). Both datasets display the characteristic diffraction features of the α-phase perovskite with a preferential crystal orientation, along with a minor PbI_2_ phase. Notably, every diffraction feature recorded with the microbeam is visibly sharper [Fig. 1 ▸(*d*)] than its counterpart obtained with the larger beam [Fig. 1 ▸(*c*)], signaling a substantial mitigation of footprint broadening.

To quantitatively assess the impact of footprint reduction on diffraction quality, we analyze the diffraction peaks corresponding to the CsFAMAPbI_3_ (001) reflection at *q* ≃ 10.0 nm^−1^ and the PbI_2_ impurity phase at *q* ≃ 9.0 nm^−1^. As shown in Fig. 1 ▸(*f*), both peaks appear significantly sharper, more symmetric, and closely approach an ideal Gaussian profile in contrast to their corresponding counterparts in Fig. 1 ▸(*e*). Critically, the FWHM of the CsFAMAPbI_3_ (001) peak decreases notably from 0.462 nm^−1^ [Fig. 1 ▸(*e*)] to 0.098 nm^−1^ [Fig. 1 ▸(*f*)], underscoring the dramatic enhancement in spectral resolution achieved via footprint minimization. Importantly, the broader, asymmetric peak shapes observed at BL14B1 [Fig. 1 ▸(*e*)] arise from a convolution of two factors: (i) non-Gaussian flux density distribution across the elongated footprint, and (ii) spatial inhomogeneity in the perovskite film’s microstructure, as further illustrated in Fig. S2. This coupling of instrumental and sample-related inhomogeneity effects leads to peak distortions, including apparent peak splitting in the PbI_2_ signal as well as an irregular asymmetry in the CsFAMAPbI_3_ (001) peak. Such distortions not only complicate the interpretation of GIWAXS data but also risk introducing unwanted errors in peak fitting and structural analysis (Qin *et al.*, 2021 ▸). For example, the splitting of the PbI_2_ peak in Fig. 1 ▸(*e*) could easily be mistaken for a secondary phase. Moreover, footprint broadening often merges adjacent diffraction peaks, making it difficult to distinguish between structurally similar phases, such as tetragonal and cubic perovskites (Yalcinkaya *et al.*, 2022 ▸; Steele *et al.*, 2023 ▸). In particular, studies aiming to resolve depth-dependent microstructural variations via GIWAXS measurements at different incident angles must be approached with caution: changes due to footprint variations with varying incidence can masquerade as spurious depth-sensitive structural transitions. While this phenomenon is also present in µ-GIWAXS, the absolute footprint lengths are approximately two orders of magnitude smaller, making this effect considerably less critical but not entirely negligible. In contrast, the µ-GIWAXS data exhibit no discernible peak splitting artifacts in either the perovskite or PbI_2_ reflections, and deliver more symmetric peak profiles (asymmetry factor closer to 1.0) that closely follow a Gaussian line shape (adjusted *R*^2^ > 0.99; see Table S1). These well defined peak profiles, with spurious modulations largely eliminated, significantly simplify data analysis.

**Figure 2:**
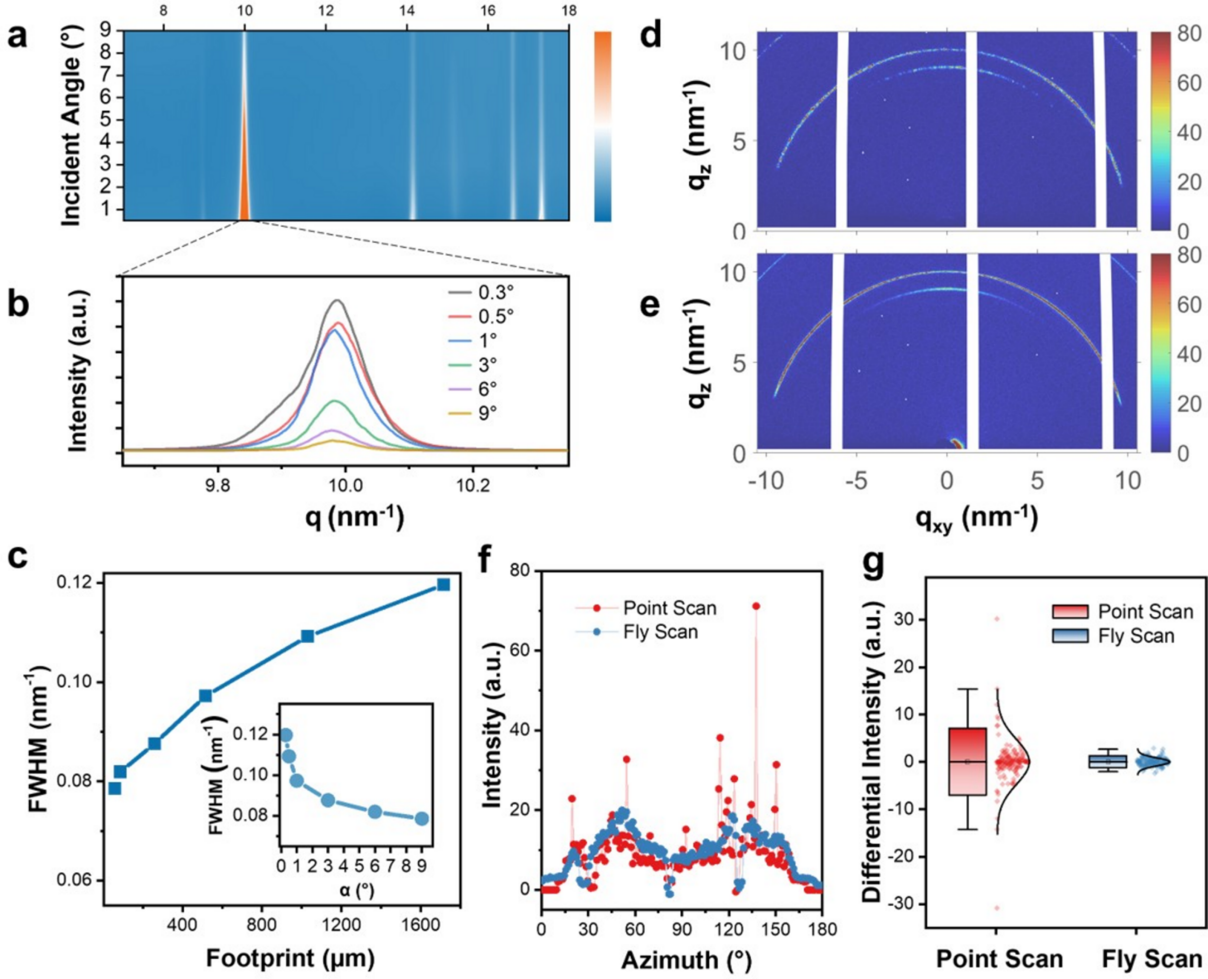
(*a*) Waterfall plot showing 1D µ-GIWAXS spectra by the azimuthal integration for 7.5 nm^−1^ ≤ *q* ≤ 18 nm^−1^ at varying incident angles measured from an MAPbI_3_ film. (*b*) 1D integrated µ-GIWAXS profiles of the MAPbI_3_ (110) peak at varying incident angles. (*c*) Stitched 2D diffraction pattern from the same perovskite film measured at different grazing incident angles. (*d*) Correlation between the MAPbI_3_ (110) diffraction peak FWHM and the incident angles as well as their corresponding footprint lengths. 2D µ-GIWAXS patterns obtained using (*e*) fixed-point and (*f*) fly scanning modes, respectively. (*g*) Radially integrated intensity plots along the MAPbI_3_ (110) diffraction ring, measured at a fixed point and by beam-direction-perpendicular fly scanning. (*h*) Statistical distribution of the curve differences calculated from (*g*).

To further elucidate how geometric footprint effects constrain reciprocal-space resolution, we performed µ-GIWAXS measurements on an MAPbI_3_ film by varying the incident angle at the BL03HB beamline. The FWHM of the MAPbI_3_ (110) diffraction peak from the 1D integrated GIWAXS was observed to change accordingly [Figs. 2 ▸(*a*)–2(*c*)]. With an X-ray beam longitudinal size of approximately ∼2.9 µm, the footprint on the sample ranges from 553.9 µm at an incident angle of 0.3° to 18.5 µm at 9°, with the corresponding FWHM of the (110) diffraction peak decreasing from 0.120 nm^−1^ to 0.079 nm^−1^, as shown in Figs. 2 ▸(*a*) and 2(*b*). Fig. 2 ▸(*c*) presents a stitched image of diffraction patterns for perovskite (110) at various grazing incident angles, intuitively showing the reduction of the diffraction ring width with increasing grazing angle. By correlating the grazing incident angle, X-ray footprint length and the FWHM values [shown in Fig. 2 ▸(*d*)], we can further quantitatively illustrate the footprint effects on diffraction peak broadening. Specifically, as the grazing incident angle increases from 0° to 3°, the FWHM of the diffraction peaks decreases significantly, and, beyond 3°, the reduction in FWHM slows down. There is an almost linear relationship between the footprint length and the FWHM of the diffraction peaks, which is consistent with the established geometrical broadening models (Fig. S3): the projection *D* on the detector scales proportionally with the footprint Δ when footprint-induced broadening is the dominant instrumental contribution. By extrapolating the curve in Fig. 2 ▸(*d*) to zero footprint length, the intrinsic peak width of the present µ-GIWAXS setup at BL03HB can be estimated, which represents the combined contribution of microstructural broadening and the residual non-footprint instrumental terms (beam divergence, detector PSF and energy bandwidth). This footprint-corrected width can serve as the input for quantitative line-profile analysis such as Scherrer grain-size estimation and Williamson–Hall strain-size separation (Smilgies, 2009 ▸; Williamson & Hall, 1953 ▸). We emphasize that peak positions and derived lattice parameters remain largely robust against footprint broadening, preserving the quantitative reliability of standard GIWAXS for phase identification and*d*-spacing determination.

The unique advantages of µ-GIWAXS are most pronounced at very small incidence angles (*e.g.* 0.05°), as the footprint of the standard beam becomes overwhelmingly severe. However, the simple geometric model (Δ = *d*/sinα) becomes insufficient in this context, where refraction, Yoneda enhancement and the sharp transition in penetration depth complicate the footprint-broadening interpretation (Yoneda, 1963 ▸; Steele *et al.*, 2023 ▸). The incidence angles employed in this study (0.3–9°) are all above α_c_ (∼0.22°), ensuring the validity of the geometric model.

Equivalent footprint lengths can be achieved using different combinations of beam size and incidence angle, but with fundamentally different depth sensitivities. For instance, a footprint of ∼5.7 mm corresponds to the large beam (∼300 µm) at 3° or the microbeam (∼3 µm) at ∼0.03°—the latter below α_c_ and thus confining the probe to the near-surface evanescent region. µ-GIWAXS therefore uniquely enables simultaneous minimization of footprint broadening and enhancement of surface sensitivity.

One thing that must be considered is the µ-GIWAXS data acquisition strategy. It is well known that the statistical reliability of XRD data from polycrystalline materials deteriorates with reduced beam size, as the interaction volume between X-rays and the material contains an insufficient number of crystallites to ensure statistically representative sampling. Therefore, special caution must be exercised when collecting µ-GIWAXS data. To address this, we propose fly scanning, which enlarges the X-ray–material interaction volume while preserving spectral resolution. This is achieved by continuously translating the sample stage back and forth perpendicular to the X-ray beam. Specifically, during exposure, the stage moves uniformly along the *y*-direction with a lateral scanning range of 1 mm (as illustrated in Fig. S4). The GIWAXS patterns obtained through fixed point measurement [Fig. 2 ▸(*e*)] and fly scanning [Fig. 2 ▸(*f*)] demonstrate almost identical peak widths for MAPbI_3_ (110) rings (FWHM of 0.109 nm^−1^ and 0.113 nm^−1^, respectively), confirming preserved spectral resolution (Fig. S5). Notably, diffraction rings in Fig. 2 ▸(*e*) exhibit less continuous rings than those in Fig. 2 ▸(*f*), evidencing improved data statistics. The improvement by fly scanning becomes particularly pronounced in their corresponding radially integrated intensity plots along the MAPbI_3_ (110) ring in Figs. 2 ▸(*e*) and 2 ▸(*f*). Through differential analysis of these plots (Fig. S6), we derived azimuthal intensity differential distribution curves [Fig. 2 ▸(*h*)], which clearly reveal significantly reduced orientation dispersion achieved via fly scanning. Therefore, this strategy preserves spectral resolution and enhances data statistics by increasing the number of sampled crystallites. Furthermore, it minimizes X-ray irradiation time at individual positions, thereby mitigating radiation damage per unit area—a critical consideration in µ-GIWAXS measurements. The fly scanning inherently averages the diffraction signal over a larger lateral area, which may smooth out local structural variations. Therefore, fly scanning is better suited for obtaining statistically representative information (*e.g.* accurate orientation distribution), whereas fixed-point or fine-step scanning is preferable when spatial resolution is the primary concern.

### 3.2. Irradiation effect of microbeam

Prolonged X-ray irradiation is known to cause radiation damage in perovskites, leading to observable microstructural changes (Kirmani & Sellers, 2025 ▸; Stuckelberger *et al.*, 2020 ▸). To achieve an X-ray microbeam, the beamline acceptance angle must be reduced, which inevitably decreases X-ray flux. Although the total flux is reduced, the smaller beam size results in significantly higher flux density. For instance, the flux density at BL03HB is estimated to be about three orders of magnitude higher than at BL14B1, potentially exacerbating radiation damage. While the protective role of nitro­gen is recognized for conventional GIWAXS, it remains a question as to whether this protection holds under the extreme conditions of µ-GIWAXS (Francisco *et al.*, 2024 ▸; Svanström *et al.*, 2021 ▸). Therefore, evaluating the microbeam irradiation effects on perovskite films during µ-GIWAXS measurements becomes essential. Additionally, the presence of moisture and oxygen during experiments must be considered, as these factors may accelerate perovskite film decomposition (Zhu *et al.*, 2023 ▸; Zhang & Han, 2020 ▸). To systematically investigate these effects, µ-GIWAXS measurements with an X-ray incidence angle of 0.5° were conducted continuously on the same spot of pure MAPbI_3_ and CsFAMAPbI_3_ films, both in air and nitro­gen environments. Each µ-GIWAXS pattern was acquired with 30 s irradiation, with total measurement durations of 60 min in each case. Throughout the experiments, the beamline hutch was maintained at ∼20°C and ∼30% relative humidity.

**Figure 3:**
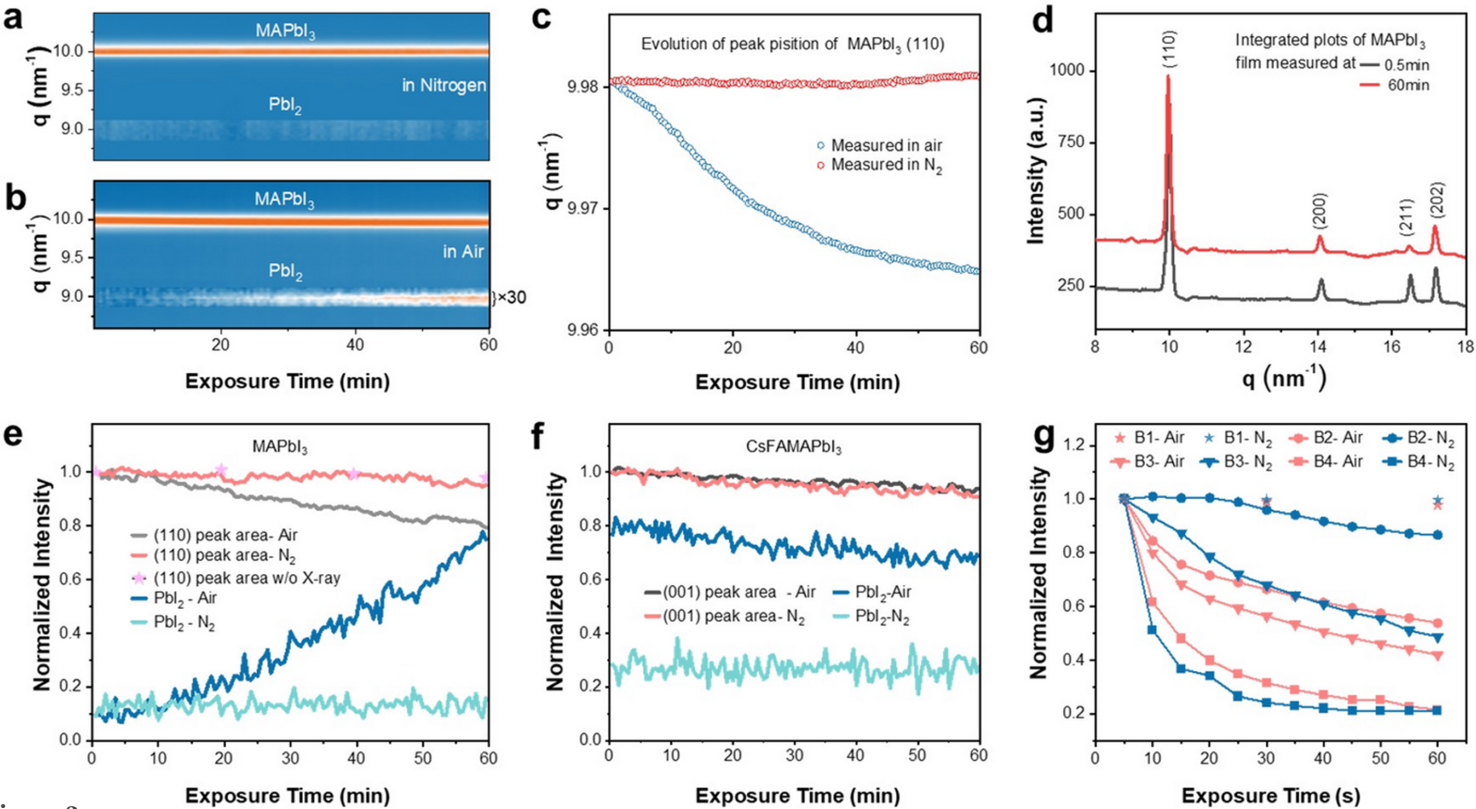
(*a*, *b*) Contour plots of the µ-GIWAXS 1D intensity profile as functions of X-ray irradiation time for MAPbI_3_ films in (*a*) nitro­gen and (*b*) air. (*c*) The MAPbI_3_ (110) diffraction peak position as functions of time derived from (*a*) and (*b*). (*d*) The µ-GIWAXS 1D intensity profile of the film measured in air at the beginning and at 60 min. (*e*) The evolution of MAPbI_3_ (110) and PbI_2_ peak intensity for the MAPbI_3_ film under X-ray irradiation in air and nitro­gen environments. (*f*) The corresponding CsFAMAPbI_3_ (001) and PbI_2_ peak intensity variations for the CsFAMAPbI_3_ film under identical irradiation conditions. To enhance the visual clarity, the PbI_2_ content (*q* = 8.87–9.12 nm^−1^) has been magnified 30 times in (*a*), (*b*), (*e*) and (*f*). (*g*) The CsFAMAPbI_3_ (001) peak intensity as a function of irradiation time at beamlines BL03HB and BL17UM, with flux density increased from the lowest (B1) to the highest (B4).

Contour plots of the µ-GIWAXS 1D intensity profile as functions of X-ray irradiation time for MAPbI_3_ films measured in nitro­gen [Fig. 3 ▸(*a*)] and in air [Fig. 3 ▸(*b*)] reveal their structural evolution during irradiation. In a nitro­gen environment, the MAPbI_3_ (110) diffraction peak maintains mostly constant intensity with no detectable PbI_2_ formation. In contrast, in ambient air, progressive attenuation of the MAPbI_3_ (110) peak concurrent with PbI_2_ signal accumulation is observed. Notably, the time-dependent (110) peak position drift occurs exclusively in air. Quantitative analysis of these phenomena is present in Figs. 3 ▸(*c*)–3(*f*). In Fig. 3 ▸(*c*), the MAPbI_3_ (110) peak in air shifts from 9.980 to 9.965 nm^−1^ during 60 min irradiation, with no equivalent shift detected in nitro­gen. Moreover, Fig. 3 ▸(*d*) displays µ-GIWAXS 1D integrated plots for the MAPbI_3_ film in air at initial and final stages, with the corresponding µ-GIWAXS patterns shown in Fig. S7. In addition to the MAPbI_3_ (110) peak intensity drop and position shift, the MAPbI_3_ (211) peak located at *q* = 16.5 nm^−1^ severely decreases. The observed peak shift could align with the reported tetragonal-to-cubic phase transitions induced by thermal effects, proposing localized X-ray heating as a potential driver for this structural transformation. However, intermittent measurements of the irradiated MAPbI_3_ film at 20 min intervals (Fig. S8) reveal non-reversible (110) peak position changes, conclusively demonstrating that the shift originates from irradiation-induced decomposition in air rather than thermally activated reversible phase transitions. Moreover, the quantitative variation of diffraction peak intensities under irradiation reveals distinct degradation pathways for MAPbI_3_ in different environments. As shown in Fig. 3 ▸(*e*), the (110) peak of MAPbI_3_ in air decreases nearly linearly with irradiation time, accompanied by a continuous rise in the PbI_2_ peak intensity, indicating progressive decomposition of MAPbI_3_ into PbI_2_. In contrast, the same perovskite phase in N_2_ exhibits negligible degradation with no detectable PbI_2_ formation. To verify the origin of the intensity variation in air, we further conducted a µ-GIWAXS measurement on another MAPbI_3_ film every 20 min in air. Fig. 3 ▸(*e*) shows that the stable MAPbI_3_ (110) peak intensity throughout the measurement indicates that ambient conditions alone do not trigger rapid MAPbI_3_ degradation. Thus, the rapid decomposition observed in air arises from the synergistic effect of X-ray irradiation and environmental factors (*e.g.* moisture/oxygen), as neither factor alone induces significant degradation.

For CsFAMAPbI_3_, known for its enhanced environmental stability, the contour plots of the µ-GIWAXS 1D intensity profile as functions of X-ray irradiation time in both N_2_ and air (Fig. S9) show no pronounced decline in the (001) peak intensity. However, Fig. 3 ▸(*f*) reveals a gradual decomposition of the α-phase (001) peak in both environments, albeit with distinct PbI_2_ evolution trends: the PbI_2_ content remains constant in N_2_ but slightly decreases in air despite ongoing perovskite decomposition. This phenomenon can be explained by PbI_2_ from the decomposition of CsFAMAPbI_3_ decomposed further by X-rays into iodine and metallic Pb (Pb^0^) (Dang *et al.*, 2017 ▸). It should be noted that excess PbI_2_ and metallic Pb are known to exacerbate hysteresis in perovskite photovoltaic devices (Lin *et al.*, 2021 ▸; Zhao *et al.*, 2022 ▸). Therefore, one must be aware of the decomposition of that PbI_2_ in the investigation of irradiation effects of perovskites. In the MAPbI_3_ films with minimal amount of PbI_2_, X-ray irradiation in air predominantly drives MAPbI_3_-to-PbI_2_ decomposition, with PbI_2_ content increasing linearly over time [Fig. 3 ▸(*e*)]. Prolonged irradiation may eventually activate PbI_2_ photodecomposition, offsetting its accumulation from perovskite degradation. To demonstrate the importance of PbI_2_ decomposition under X-ray irradiation, Fig. S10 tracks the (001) peak of an FAPbI_3_ film with 10% excess PbI_2_ under X-ray irradiation. Despite accelerated FAPbI_3_ decomposition in air, PbI_2_ content concurrently decreases (the degradation of PbI_2_ itself can outpace its generation) whereas, in N_2_, slower perovskite degradation coincides with stable PbI_2_ levels. These observations demonstrate that X-ray induced PbI_2_ decomposition is not negligible in both environments.

The irradiation-induced degradation of perovskite films exhibits a strong dependence on photon flux density, a critical factor requiring systematic analysis. Experiments were primarily conducted at the BL17UM beamline, which provides higher photon flux and a smaller beam spot compared with BL03HB. By employing an aluminium attenuator, different photon flux densities can be achieved at the sample spot. Fig. 3 ▸(*g*) summarizes the evolution of the perovskite diffraction peak intensity from CsFAMAPbI_3_ films both in air and N_2_ under four distinct flux densities at BL03HB and BL17UM (B1 to B4, see Table S2). Notably, at the highest flux density (B4: 6.6 × 10^20^ photons s^−1^ cm^−2^), the peak intensity decreases by ∼50% within 10 s even in N_2_, indicating severe X-ray-induced decomposition. A clear trend emerges: higher flux densities accelerate degradation across both environments. To quantify irradiation-driven decomposition kinetics, all curves in Fig. 3 ▸(*g*) were fitted with an exponential decay model, yielding the characteristic time constants (τ) listed in Table S2. From Fig. 3 ▸(*g*) and Table S2, it can be concluded that a photon flux density of the order of ≤10^15^ photons s^−1^ cm^−2^ has a negligible impact on CsFAMAPbI_3_ films. However, with a photon flux density of over ∼10^17^ photons s^−1^ cm^−2^, the CsFAMAPbI_3_ films suffer noticeable decomposition after just 1 min of continuous irradiation. Since radiation damage is directly proportional to the absorbed dose (measured in Gray) and considering the significant variations in both X-ray energy and photon flux inherent to synchrotron experiments, we quantified this critical parameter through dose calculations. The absorbed dose in the thin films was computed using the formula 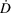 = 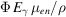, with the detailed calculation process provided in Note S1 in the supporting information (Berejnov *et al.*, 2021 ▸). The absorbed dose rate in Mode B1 was calculated to be 1.25 × 10^6^ Gy s^−1^, yielding a total dose of approximately 10^8^ Gy after 1 h of irradiation. This flux-dependent behavior underscores the necessity of optimizing irradiation parameters for perovskite characterization to minimize beam-induced artifacts. In radiation-sensitive applications, such as perovskite photovoltaics for space-based solar panels or X-ray detectors, the radiation tolerance of perovskite semiconductors is obviously a critical operational criterion, which requires further rigorous and comprehensive investigation. As for the µ-GIWAXS measurements, measuring the sample at a fixed point for a prolonged time should be avoided (Wei & Huang, 2019 ▸; Tu *et al.*, 2021 ▸; Kirmani *et al.*, 2022 ▸). Based on the present study, an inert nitro­gen atmosphere can effectively mitigate irradiation-induced degradation in perovskite samples when the absorbed dose rate remains at or below 10^6^ Gy s^−1^ at an X-ray energy of 10 keV. For comparison, da Silva *et al.* reported observable damage above ∼10^4^ Gy under 8.5 keV nano-beam condition (Francisco *et al.*, 2024 ▸). The comparatively higher tolerance in the present work is likely attributable to the harder X-ray energy (10 keV, below the Pb *L*-edges), which results in a lower photoelectric absorption cross-section. These dose thresholds are not universal material constants; they depend on beam energy, dose rate, incidence angle, film composition and thickness, and atmospheric environment. The values reported here are therefore specific to the present experimental conditions and serve as practical reference points for µ-GIWAXS experiments on halide perovskite thin films under comparable configurations.

Beyond photon flux density, perovskite film decomposition is also strongly influenced by the X-ray incidence angle during measurements. The relationship between decomposition and incidence angle is highly non-linear, governed by two competing factors: beam footprint (which determines the illuminated area) and X-ray penetration depth (where intensity attenuates to 1/*e* of the initial value) (Steele *et al.*, 2023 ▸; Francisco *et al.*, 2024 ▸). At smaller incidence angles (α_i_ < α_c_), total external reflection confines the beam to a shallow surface layer (several nm). Despite intensity enhancement at the interface, the absorbed dose remains low due to minimal interaction volume. As α_i_ increases toward and beyond α_c_, the beam footprint decreases proportionally to 1/sin(α_i_), concentrating the photon flux over a smaller area and increasing the photon density per unit area. Simultaneously, the X-ray penetration depth increases sharply, enabling deeper inter­action and greater energy deposition throughout the film thickness. The combined effect of reduced footprint and increased penetration depth leads to maximum absorbed dose in this regime. When α_i_ further increases, X-rays fully penetrate the film and reach the substrate. Although the reduced beam footprint leads to higher local photon density, the smaller irradiated volume results in a lower total absorbed X-ray dose within the perovskite film. Taking all these factors into account, the simplified process of the above different situations is illustrated in Table S3.

### 3.3. µ-GIWAXS mapping

Solution-processed perovskite films are prone to phase segregation and compositional inhomogeneity, which is particularly critical for large-area device fabrication (Gao *et al.*, 2024 ▸; Li *et al.*, 2018 ▸). These structural imperfections critically compromise material stability, severely degrading the performance and operational lifetime of perovskite-based devices. While conventional GIWAXS is extensively employed for microstructure characterization at macroscopic scales, its utility in resolving localized crystalline heterogeneity is inherently limited due to its large beam spot sizes, which is further elongated by the footprint effect along the incident direction (Epp, 2016 ▸). By contrast, µ-GIWAXS is able to achieve spatially resolved structural analysis through planar raster scanning, achieving micrometre-scale spatial resolution (tens of micrometres) by sequentially collecting diffraction patterns from distinct sample regions.

**Figure 4:**
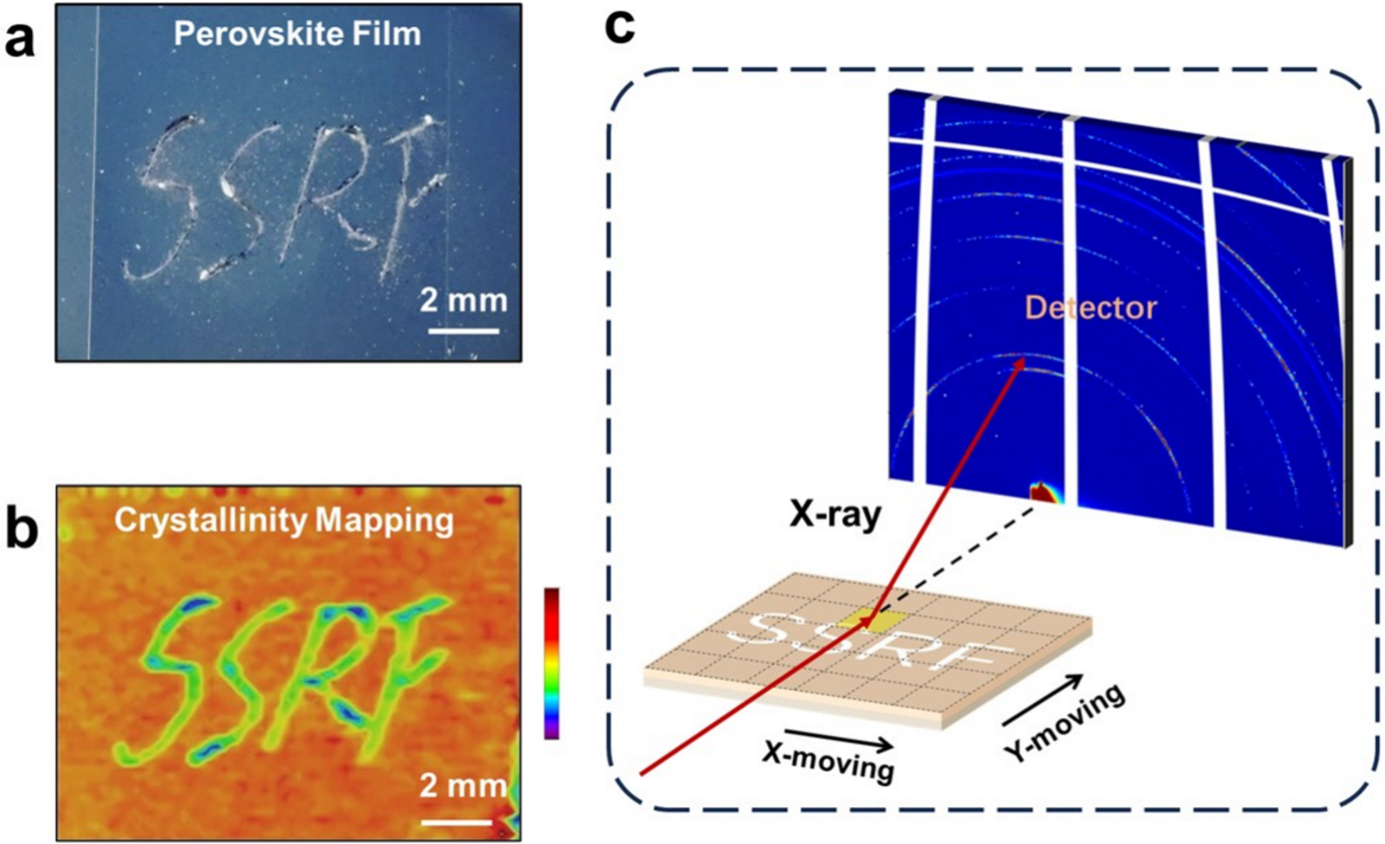
(*a*) A CsFAMAPbI_3_ film sample, inscribed with the letters ‘SSRF’, as a sample for micro-structural analysis. (*b*) Schematic of the setup for the µ-mapping experiment. (*c*) The obtained µ-GIWAXS mapping image of the CsFAMAPbI_3_ (001) diffraction peak intensity from the CsFAMAPbI_3_ film.

To validate the spatial resolution of µ-GIWAXS mapping, a 13 mm × 15 mm planar scan was performed on a CsFAMAPbI_3_ film patterned with ‘SSRF’ letters [Fig. 4 ▸(*a*)]. The experimental setup, including schematic and photographic illustrations of the scanning configuration, is reported in Fig. 4 ▸(*b*) and Fig. S11, respectively. Sample positioning was controlled using two stepper motors along two in-plane translation axes as illustrated in Fig. 4 ▸(*b*): the *X*-axis, oriented perpendicular to the incident beam (lateral direction), and the *Y*-axis, oriented approximately parallel to the incident beam (longitudinal direction). The scanning was performed row by row in a left-to-right pattern along the *X*-direction. After completing a full row of 80 points, the sample stage was incremented stepwise in the *Y*-direction (from bottom to top) before moving to the start of the subsequent row, tracing a zigzag trajectory. It should be noted that the motors for both axes move strictly within the sample plane to prevent changes in the sample-to-detector distance and the resulting peak shifts in the diffraction patterns. Throughout the scanning process, the incident angle was consistently maintained at 3°. This angle balances spatial resolution and measurement reliability: at smaller grazing angles, the elongated footprint would not only smooth out the local microstructural variations targeted in this study but also cause overlap between adjacent scanning positions, resulting in repeated X-ray exposure and potential irradiation-induced degradation. The entire scan consisted of 80 rows with 50 points per row, and each point was exposed for 5 s. From the acquired µ-GIWAXS dataset, the intensity distribution of the CsFAMAPbI_3_ (001) diffraction peak was reconstructed into the spatial mapping shown in Fig. 4 ▸(*c*). The ‘SSRF’ pattern is clearly resolved in the mapping image, corresponding to regions where mechanical scratching during patterning severely degraded perovskite crystallinity. The perfect correspondence between the optical micrograph [Fig. 4 ▸(*a*)] and the µ-GIWAXS mapping [Fig. 4 ▸(*c*)] conclusively demonstrates the technique’s capability for in-plane microstructure analysis with micrometre-scale spatial resolution.

**Figure 5:**
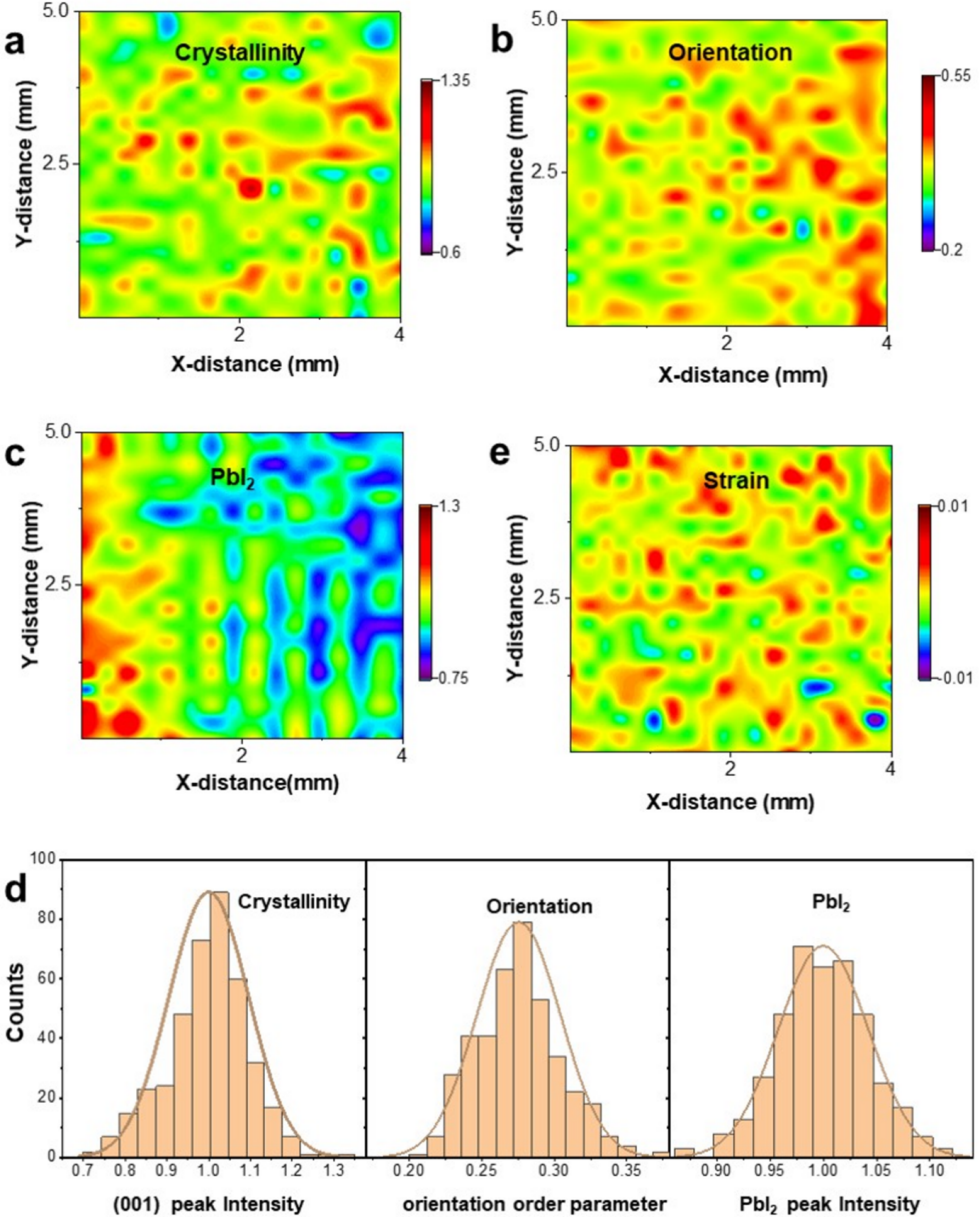
(*a*, *b*, *c*) Maps over a 4 mm × 5 mm area of a CsFAMAPbI_3_ film showing (*a*) (001) diffraction peak intensity, indicating the crystallinity of the α-phase perovskite; (*b*) (001) orientational order parameter, representing the degree of deviation from the out-of-plane orientation; and (*c*) PbI_2_ peak intensity. The statistical distributions derived from (*a*, *b*, *c*) is shown in (*d*). (*e*) Lattice strain maps over scanning areas of 4 mm × 5 mm of the CsFAMAPbI_3_ film.

To further demonstrate the versatility of µ-GIWAXS mapping, we analyzed a 4 mm × 5 mm area using a 20 × 20 point grid, corresponding to step sizes of 200 µm and 250 µm, respectively. The scanning direction, X-ray incident angle, exposure time per point, and other experimental parameters were consistent with those described in the previous section. From the µ-GIWAXS datasets, spatially resolved mappings were reconstructed to visualize key microstructural parameters including CsFAMAPbI_3_ (001) peak intensity, PbI_2_ peak intensity and CsFAMAPbI_3_ (001) orientation order parameter as shown in Figs. 5 ▸(*a*)–5(*c*).

As shown in Fig. 5 ▸(*a*), the CsFAMAPbI_3_ (001) peak intensity displays a highly heterogeneous spatial distribution, revealing spatially varying crystallization degree across the perovskite film surface. The peak intensity can differ by two times at different areas with interconnected high-intensity domains, which can be attributed to stochastic nucleation dynamics inherent to solution-processing. Fig. 5 ▸(*b*) maps the spatial distribution of orientation order parameters within the perovskite thin films. Given that crystallographic alignment dictates critical optoelectronic performance metrics, such as carrier mobility and grain boundary density, the spatial mapping of crystal orientation provides valuable information for understanding local variations in device-relevant film properties (Li, Shen *et al.*, 2023 ▸; Li *et al.*, 2024 ▸). The orientation order parameter specifically quantifies angular deviation from the (001) preferential orientation (the calculation follows established crystallographic formalism) (Song *et al.*, 2020 ▸). The enhanced diffraction intensity on the film’s right side correlates with a superior (001) orientation, which promotes the vertical transport of charge carriers. Conversely, the green regions in the map indicate a stronger (111) orientation, which is known to mitigate ion migration and enhance environmental stability. Comparison of Figs. 5 ▸(*a*) and 5 ▸(*b*) reveals that these two parameters—crystallinity and orientation—exhibit spatial inhomogeneity with distinct distributions, indicating that high crystallinity is not exclusively tied to a specific orientation. Notably, a left-to-right intensity gradient is observed in Fig. 5 ▸(*c*), differing from the stochastic heterogeneity of other parameters. This pattern may be related to the scanning trajectory, where the elongated beam footprint could accumulate irradiation damage from the initial exposure area, causing irradiation-induced decomposition of the perovskite into PbI_2_. However, a detailed analysis suggests a fabrication-based origin is more plausible. Firstly, beam-footprint-driven damage would primarily produce a *Y*-direction gradient, contrary to the observed *X*-direction variation. Secondly, the decomposition extent is considered limited due to the short, non-continuous irradiation duration (≤100 s) and the material’s self-healing properties (Wang, Song *et al.*, 2024 ▸). The poor correlation between the PbI_2_ and perovskite crystallinity distributions from Figs. 5 ▸(*a*) and 5(*c*) also supports this conclusion. The observed pattern is more likely to originate from an inhomogeneous distribution of the cationic solution during the second fabrication step of perovskite film, leading to spatially incomplete conversion of PbI_2_. The statistical data for the regions scanned in Figs. 5(*a*)–5(*c*) are summarized in Fig. 5 ▸(*d*), with detailed parameters (*e.g.* mean and standard deviation) provided in Table S4. This µ-GIWAXS mapping analysis serves as a powerful quantitative tool for microstructure characterization, demonstrating statistically comparable distributions.

Solution-processed mixed-cation perovskite films typically exhibit an inhomogeneous lattice strain distribution (Xiong *et al.*, 2024 ▸). By analyzing the µ-GIWAXS diffraction dataset acquired from the same scan as Figs. 5 ▸(*a*)–5(*c*) (see Note S2 for calculation details), we mapped the corresponding strain distribution, as shown in Fig. 5 ▸(*e*). Each point in the map corresponds to Δ*q*, defined as Δ*q* = *q*_∥_ − *q*_⊥_, where *q*_∥_ and *q*_⊥_ represent the in-plane and out-of-plane scattering vectors, respectively. Due to the inherent ‘missing wedge’ in GIWAXS, the exact in-plane and out-of-plane directions are inaccessible (Qin *et al.*, 2021 ▸). Therefore, *q*_∥_ and *q*_⊥_ were extracted from the nearest accessible azimuthal angles, yielding an approximate measure of lattice strain. A negative Δ*q* (Δ*q* < 0) signifies tensile strain, mainly resulting from the perovskite’s higher thermal expansion coefficient relative to the substrate. During cooling, the greater contraction of the perovskite imposes in-plane tension. Conversely, a positive Δ*q* (Δ*q* > 0) indicates compressive strain. Although less common, compressive strain—often linked to improved long-term stability—can be introduced via specific processing routes. The strain map reveals a non-uniform spatial distribution of tensile and compressive strains within the film, challenging the conventional assumption that only tensile strain is present. This heterogeneity may be attributed to multiple sources, including *A*-site cation size mismatch, local lattice distortions at residual PbI_2_ inclusions, compositional gradients inherent to the two-step deposition, and thermal expansion mismatch between the perovskite film and substrate (Xue *et al.*, 2020 ▸; Wang, He *et al.*, 2024 ▸; Chen *et al.*, 2019 ▸). The strain values here reflect the combined contribution of these factors; disentangling individual contributions would require more complementary spatially resolved compositional analysis (*e.g.* nano-XRF and TOF-SIMS). The co-existence of tensile and compressive strains can lead to local lattice distortions, potentially compromising the structural integrity (Zhu *et al.*, 2019 ▸). Statistical distributions of Δ*q* corresponding to Fig. 5 ▸(*e*) are provided in Fig. S12. It shows that most of the lattice points exhibit Δ*q* < 0, and the integrated value of Δ*q* is also negative, consistent with the notion that perovskite films prepared on glass substrates are dominated by tensile strain.

Finally, the influence of the scanning protocol was also evaluated. Fig. S13 presents perovskite crystallinity maps obtained from a 1 mm × 1 mm scanning area (with a 50 µm step size) and a 200 µm×200 µm area (with a 10 µm step size). The overlap between scanned points resulted in artificial continuous features, which may compromise measurement reliability. To mitigate this issue and minimize repeated X-ray irradiation, careful optimization of the X-ray incident angle and the use of a sufficiently large step size are recommended.

## 4. Conclusions

This study establishes synchrotron-based µ-GIWAXS as a transformative advancement over conventional GIWAXS by mitigating footprint effects, thereby achieving superior spectral resolution and measurement accuracy. By systematic tuning of the grazing incidence angle, we elucidated the relationship between footprint length and diffraction peak broadening, demonstrating consistency with geometrical broadening models. The implementation of beam-direction-perpendicular fly scanning further enhanced data quality while markedly reducing localized X-ray exposure.

Through systematic irradiation experiments, we evaluated the distinct degradation pathways for MAPbI_3_ and Cs_0.05_FA_0.85_MA_0.1_PbI_3_ films arising from the synergistic effect of X-ray irradiation and environmental moisture/oxygen. The concurrent photodecomposition of PbI_2_ was also identified. Under the present experimental conditions (10 keV, 0.5° incidence), a nitro­gen atmosphere effectively suppresses radiation damage at absorbed dose rates up to 10^6^ Gy s^−1^.

Furthermore, µ-GIWAXS enabled high-resolution spatial mapping of perovskite films, revealing key characteristics such as crystallinity variations, preferential orientations, PbI_2_ and lattice strain distributions. These capabilities directly address structural heterogeneity challenges in solution-processed films, particularly for large-area devices. Our findings underscore µ-GIWAXS as an indispensable tool for advancing perovskite film characterization, supporting both performance optimization and scalable manufacturing.

## 5. Related literature

The following references, not cited in the main body of the paper, have been cited in the supporting information: Rolston * et al.* (2018 ▸); Wang * et al.* (2019 ▸).

## Supplementary Material

Figs. S1 to S13, Tables S1 to S4 and Notes S1 and S2. DOI: 10.1107/S1600577526004303/vl5054sup1.pdf

## Figures and Tables

**Table 1:** Summary of the experimental configurations and optical parameters for the three synchrotron beamlines used in this study

Parameter	BL14B1	BL03HB	BL17UM
X-ray source	Bending magnet (1.27 T)	Bending magnet (2.29 T)	In-vacuum undulator
Monochromator	Si(111) DCM	Four-bounce Si(111)	Double multilayer Si(111) DCM / DMM
Micro-focusing optics	None (slit collimation)	KB mirrors	KB mirrors
Energy resolution (Δ*E*/*E*)	1.91 × 10^−4^	0.96 × 10^−4^	2 × 10^−4^
Beam size at sample (FWHM)	∼500 µm (H) × 300 µm (V)	∼5 µm (H) × 3 µm (V)	∼1 µm (H) × 0.7 µm (V)
Effective divergence at sample	∼0.25 mrad (V)	∼2.8 mrad (V)	∼1 mrad (V)
Sample positioning system	Huber 5021 six-circle diffractometer	6-DOF stage (*X*/*Y*/*Z*/pitch/yaw/roll) + 1-circle diffractometer	MD3-UP diffractometer
Detector	MarCCD225	Pilatus 2M	Eiger X 16M
Detector pixel size	73 µm × 73 µm	172 µm × 172 µm	75 µm × 75 µm

## Data Availability

The data that support the findings of this study are available from the corresponding author upon reasonable request.
