# Supplementary material for: GIWAXS using microbeam applied in halide perovskite films for high spectral resolution and mapping capability
Source: J Synchrotron Radiat. 2026 May 21;33(Pt 4):1043–54. doi: 10.1107/S1600577526004303 (PMC13344537; doi:10.1107/S1600577526004303)
Supplement: Supplementary file 1 [file s-33-01043-sup1.pdf]

JOURNAL OF SYNCHROTRON RADIATION

Volume 33 (2026)

Supporting information for article:

GIWAXS using microbeam applied in halide perovskite films for high spectral resolution and mapping capability

Meirong Fu, Bingchen He, Liujiang Zhang, Zhenhuang Su, Yanfeng Miao, Qihang Sun, Jihao Zhang, Sisheng Wang, Zhijun Wang, Xingya Wang, Yuzhu Wang, Bo Sun, Wen Wen, Yixin Zhao, Chenyue Wang and Xingyu Gao

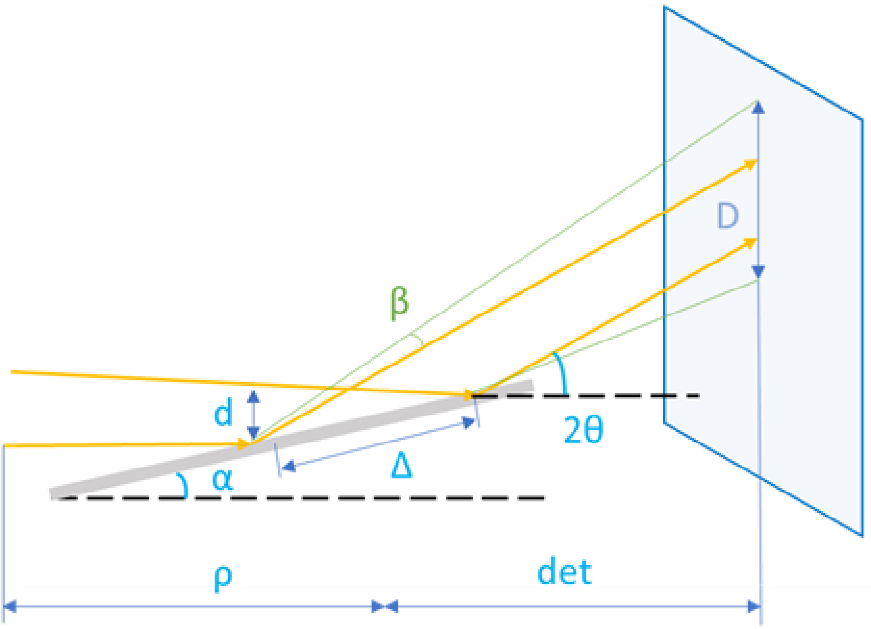

Figure S1 Schematic illustrates the broadening of diffraction peaks in GIWAXS due to footprint effects, incorporating the divergence angle of the X-ray beam. Here, β represents the deflection angle of the KB mirrors. For our GIWAXS experiments, the sample is positioned at the focal point of the KB mirrors. The distances from the KB mirrors to the sample and from the sample to the detector are denoted as ρ and det, respectively.

Based on this geometry, we have derived Equation S1 and Equation S2.

$$d = (\rho + \frac{1}{2}\,\Delta \cdot \cos\alpha) \cdot \tan(\alpha - \beta) - (\rho - \frac{1}{2}\Delta \cdot cos\alpha) \cdot \tan(\alpha + \beta) \qquad \text{Equation S1}$$

$$D = \left(\det + \frac{1}{2}\,\Delta \cdot \cos\alpha\right) \cdot \tan(2\theta + \beta) - (\det - \frac{1}{2}\,\Delta \cdot \cos\alpha) \cdot \tan(2\theta - \beta) \qquad \text{Equation S2}$$

During GIWAXS measurements, the sample is placed at the focal point of the beam, where the spot size d can be considered fixed (with $\rho = 172\ mm$). Given that the condition $1/2\Delta \cdot cos\alpha \ll \rho$ is satisfied, the footprint Δ can be reasonably approximated by：

$$\Delta = d/sin\alpha$$

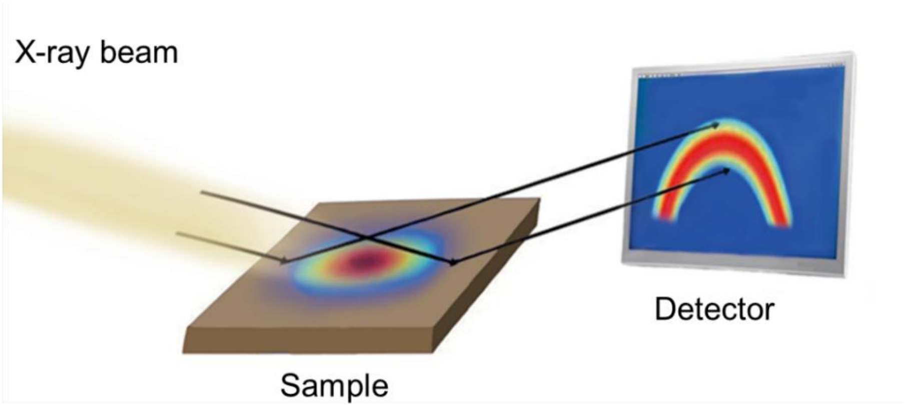

Figure S2 Schematic illustration of the broadening and distortion from a diffraction peak due to an elongated footprint, where the color distribution on the area detector represents the detected uneven-distributed diffraction intensity and that on the sample denotes the uneven diffraction intensity from the footprint due to beam flux heterogeneity across the footprint and/or local crystallographic disorder in the specimen. It should be noted that the microstructural heterogeneities could even lead to induce lattice parameter variations that manifest as diffraction angle shifts, thereby modifying detector intensity distribution patterns beyond simple peak broadening effects.

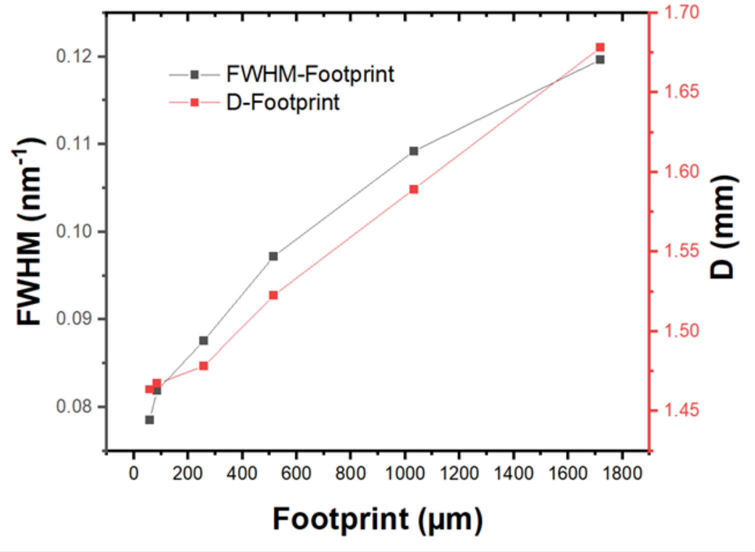

Figure S3 Variation of the (110) peak FWHM and detector beam projection (D) with footprint length measured at different incident angles.

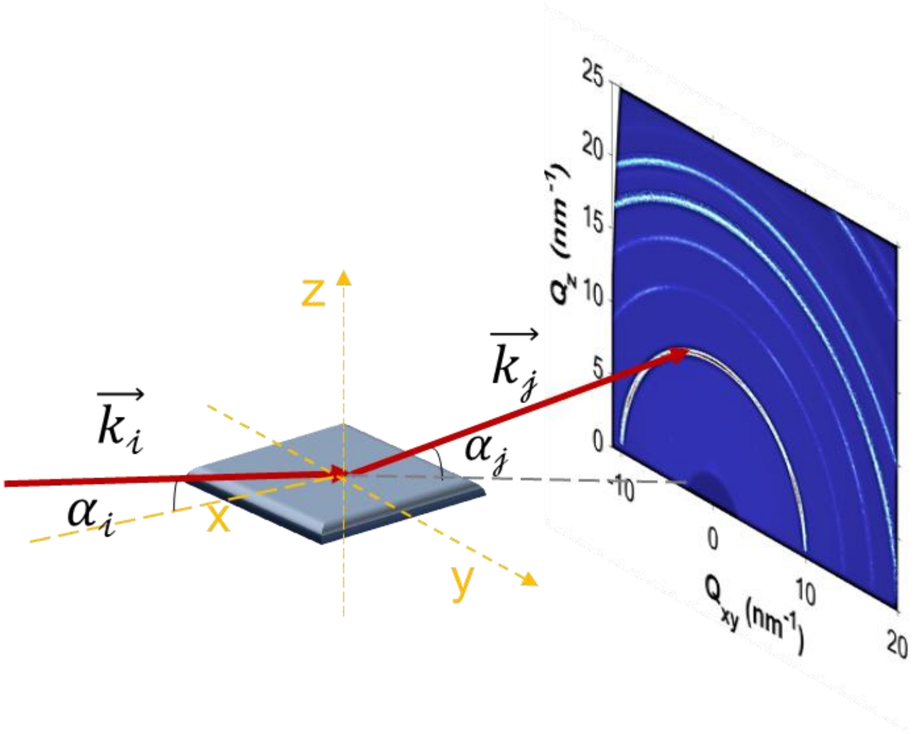

Figure S4 The schematic illustration of GIWAXS.

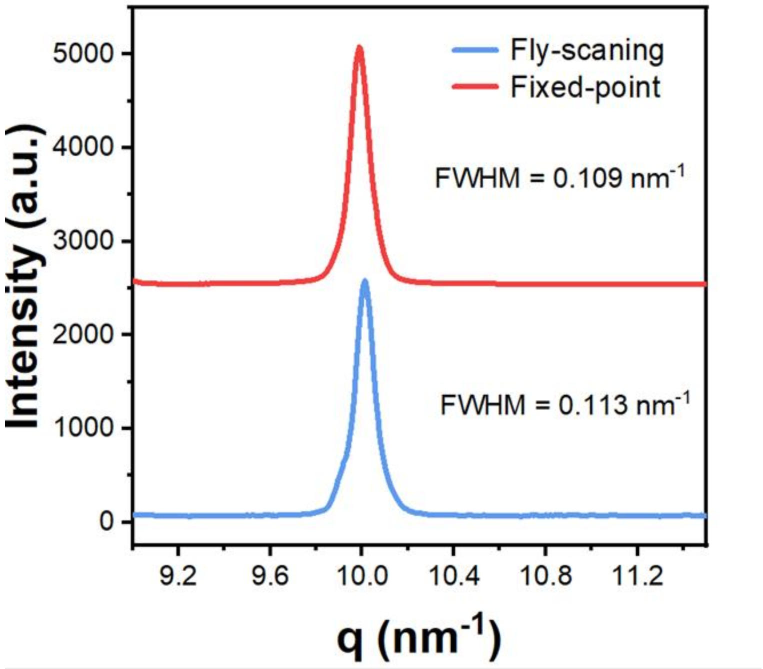

Figure S5 1D diffraction patterns of the perovskite film integrated from the Figure 2e (Fixed-point) and Figure 2f (fly-scanning), respectively.

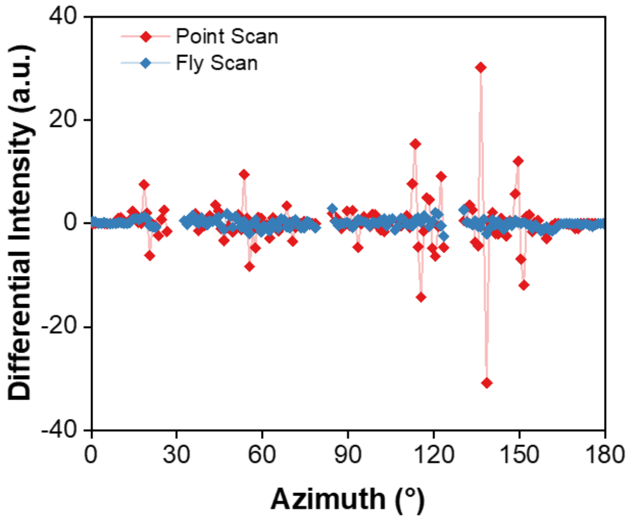

Figure S6 The differential intensity from the two different types of measurements as functions of azimuth angle derived from Figure 2g.

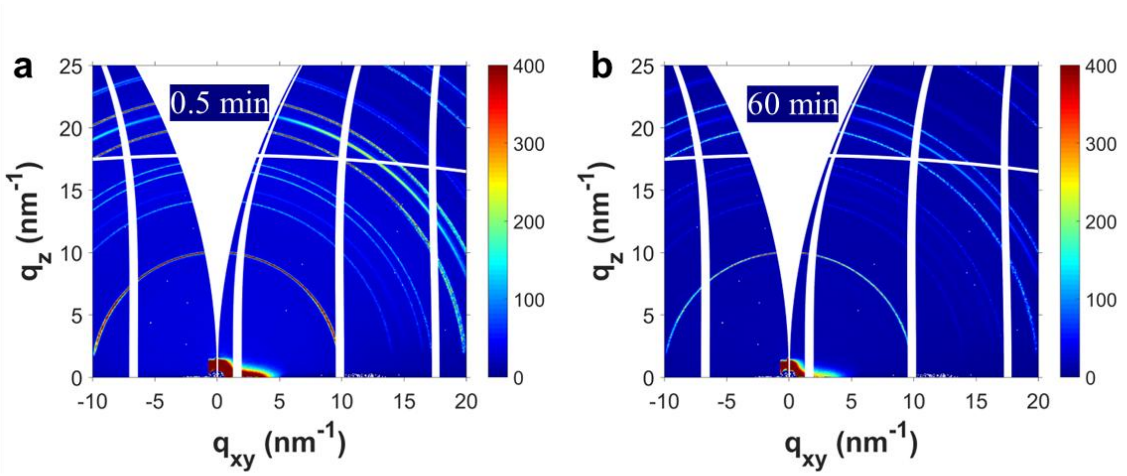

Figure S7 2D GIWAXS patterns of the $MAPbI_3$ film irradiated by X-ray in air for 0.5 min and 60 min.

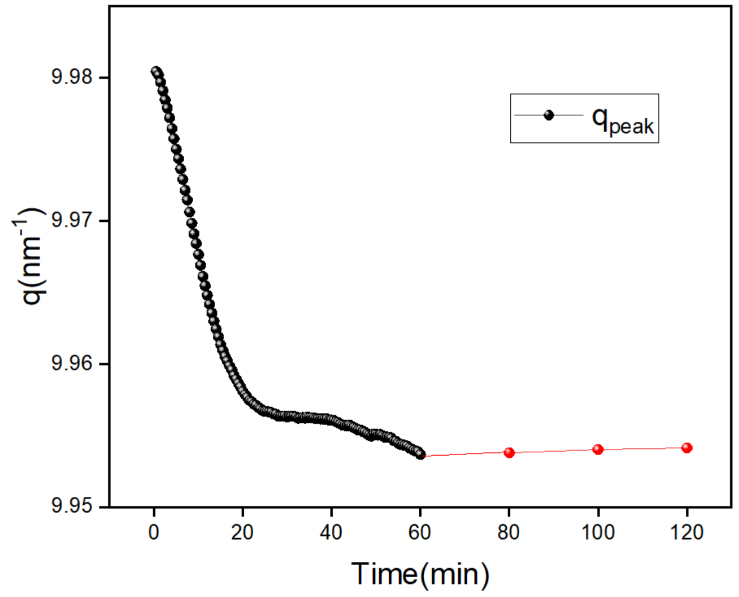

Figure S8 After subjecting the $MAPbI_3$ film to a one-hour X-ray irradiation for μ-GIWAXS measurements, we performed μ-GIWAXS tests every 20 minutes with the red dots showing the measured (110) peak position each time. All the red dots did not recover its original position before μ-GIWAXS measurements.

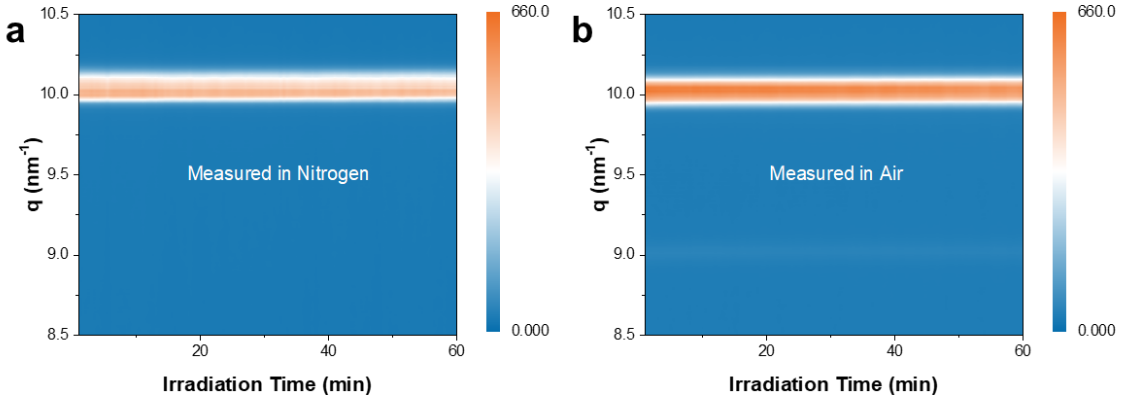

Figure S9 Contour plots of GIWAXS 1D intensity profile as functions of X-ray irradiation time for $CsFAMAPbI_3$ film measured in nitrogen (**a**) and air (**b**), respectively.

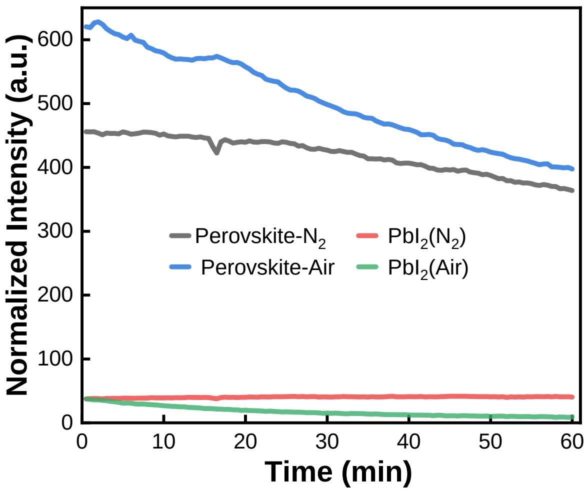

Figure S10 Evolution of $FAPbI_3$ (001) and PbI_2 (001) diffraction peak intensity under X-ray irradiation. The $FAPbI_3$ film was prepared with excess 10% PbI_2.

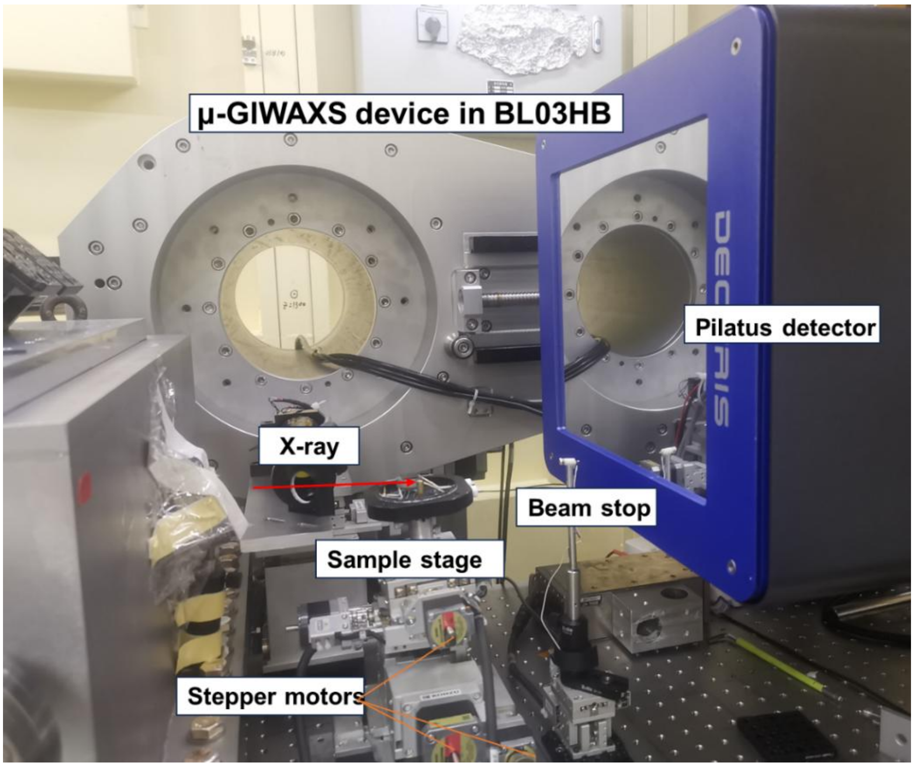

Figure S11 The experimental setup for the μ-GIWAXS at the beamline BL03HB of SSRF.

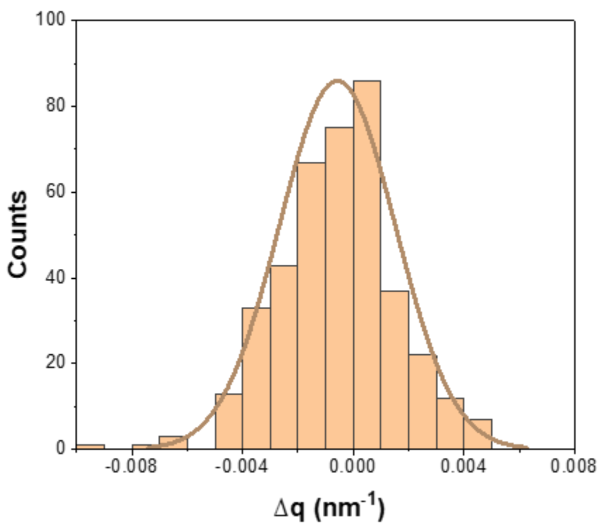

Figure S12 Statistical distributions of lattice strain in a $CsFAMAPbI_3$ perovskite film over a 4 mm × 5 mm scanning area. Here, Δq is defined as the difference between the out-of-plane and in-plane scattering vectors, $\Delta q = q_{\perp} - q_{\parallel}$.

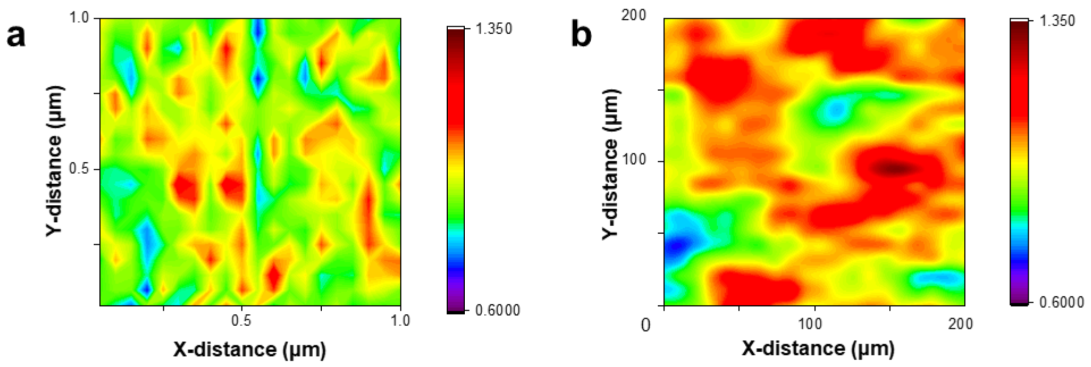

Figure S13 Perovskite crystallinity maps for (a) a 1×1 mm² scanning area with a 50 μm step size and (b) a 200×200 μm² scanning area with a 10 μm step size.

Table S1 Gaussian Fitting Parameters (adjusted R^2 and asymmetry factor) for $CsFAMAPbI_3$ (001) diffraction peaks from GIWAXS and μ-GIWAXS Data.

Parameter	GIWAXS(BL14B1)	μ-GIWAXS(BL03HB)
Gaussian fit adj. R^2	0.995	0.926
Asymmetry factor	0.875	0.812

Table S2 The parameters of X-ray beams at the 17UM and 03HB beamlines used in the present measurements, including spot size and flux intensity.

Beamline	Model name	X-ray photon flux density（@10KeV）	Decay coefficients **τ** (s)	
			In nitrogen	In air
BL03HB	B 1	7×10^{15} phs/(s·cm^2)	>2000	>2000
BL17UM	B 2	4.5×10^{17} phs/(s·cm^2)	16.93	43.59
	B 3	1.8×10^{18} phs/(s·cm^2)	15.13	39.15
	B 4	6.6×10^{20} phs/(s·cm^2)	8.82	`5.92

Table S3 The relationship of the absorbed dose of perovskite film and X-ray incident angle during GIWAXS measurements.

	$\alpha_i < \alpha_c$	$\alpha_i \approx \alpha_c$	$\alpha_i > \alpha_c$
Footprint	Large and even exceeds the film width	Decreases proportionally to $1/sin\alpha_i$	Decreases proportionally to $1/sin\alpha_i$
Penetration Depth	Very shallow (several or tens of nanometers)	Increases very sharply until it completely penetrates the film	Completely penetrates the film
Absorbed Dose	Low	Increased to its Maximum	Decreases and continues to drop as the angle increases

Table S4 The statistical parameters of the perovskite (001) peak area, PbI_2 peak area and the perovskite orientation order parameter for the scanned area of 4 mm×5 mm.

Perovskite (001) peak area		PbI_2 Peak area		perovskite orientation order parameter	
Mean	SD	Mean	SD	Mean	SD
77.94	5.89	10.87	1.38	0.36	0.033

Note S1. Calculation of Absorbed Dose

Under simplified approximation, the absorbed dose D can be estimated using the following expression[1, 2]:

$$D\,(Gy) \approx N \times E_\gamma \times \frac{f_{dep}}{m} \qquad \text{(Equation S3)}$$

where:

- D is the absorbed dose in Gray (Gy), 1Gy = 1J/kg.
- N is the total number of photons incidents on the target area.
- E_γ is the energy per photon (in joules).
- f_{dep} is the energy deposition fraction ($0<f_{dep}\leq1$), representing the proportion of incident photon energy ultimately deposited in the target volume. This factor is the most challenging to determine precisely and is highly dependent on the specific geometry and composition. In an ideal scenario of complete absorption, $f_{dep}\approx1$.
- m is the mass of the irradiated material (in kilograms).

We further derived the relationship between the absorbed dose rate, $\dot{D}$ (in Gy/s), and the photon flux density, Φ (in photons/(m^2·s)), as follows:[3]

$$\dot{D} = \Phi \cdot E_\gamma \cdot \mu_{en}/\rho \qquad \text{(Equation S4)}$$

where:

- $\dot{D}$: absorbed dose rate (Gy/s)
- Φ: photon flux density (photons/(m^2·s))

Number of photons passing through a unit area per unit time.

- μ_{en}/ρ: mass energy absorption coefficient (m^2/kg).

In cases where the photon flux density remains constant, the total absorbed dose D can be obtained by integrating the dose rate over the irradiation time t:

$$D = \int \dot{D} \cdot dt = \dot{D} \cdot t \qquad \text{(Equation S5)}$$

Under the experimental conditions described in this work, the photon energy was $E_\gamma = 10\ keV = 1.602 \times 10^{-15} J$; and the photon flux density in B1 mode was $\Phi = 7 \times 10^{19}\ phs/(m^2 \cdot s)$. The μ_{en}/ρ for the perovskite sample was found to be approximately 11.2 m^2/kg, as obtained from the NIST XCOM database. Substituting these values into the equation yields an absorbed dose rate of $\dot{D}1 \approx 1.254 \times 10^6\ Gy/s$ for the B1 mode. Under this condition, the α-phase diffraction peak area of $CsFAMAPbI_3$ retained over 90% of its initial intensity after one hour of continuous irradiation, demonstrating high radiation tolerance. Assuming a stable photon flux, the total absorbed dose is $D \approx 4.514 \times 10^8\ Gy$.

Note S2. Lattice Strain Calculation

The strain index σ of the perovskite lattice was evaluated using the 2θ–sin²ψ method based on Bragg's law and the generalized Hooke's law[4, 5], as given by Equation S3:

$$\sigma = \frac{E}{(1+\nu)sin^2\varphi} \cdot (\frac{d_\varphi - d_n}{d_n}) \qquad \text{(Equation S6)}$$

where φ and n denote scattering vector angles relative to the normal surface direction of the perovskite film. For the out-of-plane direction, φ = 90° (⊥), and for the in-plane direction, n = 0° (‖). Substituting φ = 90° into Equation S3 yields $sin^2\psi = 1$, leading to the simplified form of Equation:

$$\sigma = \frac{E}{(1+\nu)} \cdot (\frac{d_\perp - d_\parallel}{d_\parallel}) \qquad \text{(Equation S7)}$$

Using the relation $q = 2\pi/d$, Equation S4 can be rewritten in terms of the scattering vector q as:

$$\sigma = \frac{E}{(1+\nu)} \cdot (\frac{q_\parallel - q_\perp}{q_\parallel}) \qquad \text{(Equation S8)}$$

Let $\Delta q = q_{//} – q_\perp$ represent the difference between the in-plane and out-of-plane scattering vectors. Since the variation in $q_\perp$ is negligible compared to Δq, it can be approximated by a constant value q_c, resulting in Equation S6:

$$\sigma = \frac{E}{(1+\nu)} \cdot (\frac{\Delta q}{q_c}) \qquad \text{(Equation S9)}$$

Here, *E* is Young's modulus and *v* is Poisson's ratio of the perovskite film. The constant scattering vector q_c is set to 10 nm^{-1}[6]. According to Equation S6, the strain index σ is proportional to Δq. The Δq is calculated by subtracting the $q_\parallel$ in-plane scattering vector value from that ($q_\perp$) of out-of-plane. It should be noted that due to the inherent "missing wedge" in GIWAXS, the exact in-plane and out-of-plane directions are inaccessible. Therefore, the parameters mentioned above ($d_\parallel$, $d_\perp$, $q_\parallel$, $q_\perp$ and Δq) were extracted from the nearest accessible azimuthal angles, yielding an approximate measure of lattice strain.

References

1. Francisco, M. C. d. S.; Rodrigo, S.; Maria, G. D. G.; Verônica, C. T.; Ana, F. N.; Hélio, C. N. T., X-ray dose effects and strategies to mitigate beam damage in metal halide perovskites under high brilliance X-ray photon sources. *Energy Materials* **2024,** *4* (5), 400058.
2. Berejnov, V.; Rubinstein, B.; Melo, L. G. A.; Hitchcock, A. P., Calculating absorption dose when X-ray irradiation modifies material quantity and chemistry. *Journal of Synchrotron Radiation* **2021,** *28* (3), 834-848.
3. Berejnov, V.; Rubinstein, B.; Melo, L. G. A.; Hitchcock, A. P., Calculating absorption dose when X-ray irradiation modifies material quantity and chemistry. *J Synchrotron Radiat* **2021,** *28* (Pt 3), 834-848.
4. Wang, H.; Zhu, C.; Liu, L.; Ma, S.; Liu, P.; Wu, J.; Shi, C.; Du, Q.; Hao, Y.; Xiang, S.; Chen, H.; Chen, P.; Bai, Y.; Zhou, H.; Li, Y.; Chen, Q., Interfacial Residual Stress Relaxation in Perovskite Solar Cells with Improved Stability. *Advanced Materials* **2019,** *31* (48), 1904408.
5. Zhu, C.; Niu, X.; Fu, Y.; Li, N.; Hu, C.; Chen, Y.; He, X.; Na, G.; Liu, P.; Zai, H.; Ge, Y.; Lu, Y.; Ke, X.; Bai, Y.; Yang, S.; Chen, P.; Li, Y.; Sui, M.; Zhang, L.; Zhou, H.; Chen, Q., Strain engineering in perovskite solar cells and its impacts on carrier dynamics. *Nature Communications* **2019,** *10* (1), 815.
6. Rolston, N.; Bush, K. A.; Printz, A. D.; Gold-Parker, A.; Ding, Y.; Toney, M. F.; McGehee, M. D.; Dauskardt, R. H., Engineering Stress in Perovskite Solar Cells to Improve Stability. *Advanced Energy Materials* **2018,** *8* (29), 1802139.
